# Identification of measures predictive of age of puberty onset in gilts

**DOI:** 10.1093/tas/txz173

**Published:** 2019-11-14

**Authors:** Kody L Graves, Bethany R Mordhorst, Elane C Wright, Benjamin J Hale, Kenneth J Stalder, Aileen F Keating, Jason W Ross

**Affiliations:** Department of Animal Sciences, Iowa State University, Ames, IA

**Keywords:** follicle development, gilt development, puberty

## Abstract

A potential indicator of female lifetime productivity in swine is age of puberty, when a gilt achieves her first behavioral estrus. Follicular activity, as determined by tertiary follicle development, in prepubertal gilts begins during postnatal day (PND) 75 to 115. The central hypothesis of this study is that gilts demonstrating tertiary follicle development earlier in life, assessed using vulva size as a proxy, achieve puberty earlier in life compared with counterparts of a similar age and weight that lack tertiary follicle development. The objectives of this project were to identify a developmental time point when variation in ovarian development exists and to determine whether a relationship between the age prepubertal ovarian development and the age at onset of puberty exists. To accomplish this, 155 gilts of similar age (± 2 d) were weighed and vulva size measured on PND 75, 85, 95, 105, and 115. Vulva measures, including vulva width (VW), vulva length (VL), and vulva area (VA), were utilized as developmental proxies for follicular activity. At each time point, gilts (*n* = 10) were sacrificed and ovarian follicular activity recorded. In a subset of gilts (*n* = 105), estrus detection was conducted daily on PND days 126 to 200. Mean VA on PND 75, 85, 95, 105, and 115 was 596 ± 206, 683 ± 190, 864 ± 212, 1014 ± 228, and 1265 ± 252 mm^2^, respectively. Of the gilts demonstrating behavioral estrus, 28 were within PND 140 to 160, 36 between PND 161 to 180, 15 between PND 181 to 200, and 26 did not demonstrate estrus behavior within 200 d of age. All gilts euthanized at PND 75 lacked follicular activity as defined by having a minimum of 2 antral follicles per ovary, whereas 60%, 80%, 90%, and 100% demonstrated follicular activity on PND 85, 95, 105, and 115, respectively. Body weight at PND 75 and VW at PND 115 were correlated to age at first estrus (*P* < 0.05). Of the gilts whose VA was less than 1 SD from the mean on PND 95 (i.e., <652 mm^2^), 31% and 50% demonstrated their first behavioral estrus by PND 180 and 200, respectively. However, of gilts whose VA was within or greater than 1 SD of the mean (i.e., ≥652 mm^2^), 66% and 79% exhibited estrus prior to PND 180 and 200, respectively. These data support utilization of VA changes between 95 and 115 d of age as a useful tool to identify replacement gilts prior to puberty for inclusion into the sow herd.

## INTRODUCTION

Age of first estrus, or puberty onset, has a positive predictive value for future reproductive performance within a breeding herd (Culbertson and Mabry, 1995; Sterning et al., 1998; Patterson et al., 2010). Thus, this phenotype may be associated with sow lifetime productivity (SLP), or the number of quality pigs a sow produces from the time at which she becomes breeding eligible until the time at which she leaves the herd. Improvement in SLP is difficult as it is lowly heritable, likely the result of being a complex trait controlled by numerous loci (Serenius and Stalder, 2004). In addition to age of puberty onset, SLP is influenced by multiple components, such as structural conformation, number of nonproductive days, as well as pre- and post-wean environmental factors (Serenius and Stalder, 2006; Patterson et al., 2010).

Thus, a specific reproductive milestone, such as age at puberty, could represent a useful phenotype to select or reject gilts to enter the breeding herd. Furthermore, because gilts comprise a large portion of the breeding herd, their reproductive efficiency when they enter the system can greatly affect overall herd performance as well as individual lifetime performance. The potential utility of puberty, or age at first estrus, had been previously demonstrated as gilts exhibiting their first behavioral estrus early in life (<153 d of age) compared with later (154 to 180 d of age) have fewer nonproductive days in the sow herd (Patterson et al., 2010). In addition, gilts exhibiting estrus after 180 d of age had a lower service rate than gilts whose first estrus was detected prior to 180 d of age (Patterson et al., 2010). This observation has also been corroborated by others in that gilts achieving their first estrus at a younger age have an increased likelihood of producing a third parity (Engblom et al., 2008).

Numerous physical and physiological factors, including growth patterns, activation of the hypothalamic–pituitary–gonadal axis, and initial follicular development preceding puberty onset can be quite variable among a gilt cohort. Genetic selection and nutritional management to produce leaner hogs may also result in delayed puberty onset (Evans and O‘Doherty, 2001). However, growth performance may be related to puberty onset as Kummer et al. (2009) demonstrated gilts with faster growth rates at time of boar exposure achieved puberty earlier than slower growth counterparts although Patterson et al. (2002a) did not observe a similar response with respect to puberty onset when nutritionally altering lean growth rates in gilts. In relation to this, gilts with heavier body weight (BW) at time of boar exposure were younger at puberty onset (King, 1989), suggesting that rapid growth, and by result, BW at specific stages or age of development could be predictive of age of puberty.

In addition to BW, other quantifiable markers that are more directly and biologically related to factors contributing to puberty onset may be predictive of age at puberty as well if measured at the appropriate stage of a gilt’s life. We previously observed significant follicular variation in gilts sacrificed at approximately 90 to 100 d of age (Pearce et al., 2013), substantiating others observations that variation among gilts with respect to the beginning of follicular growth begins at approximately 60 to 100 d of age (Schwarz et al., 2008). Our observation included that those gilts with greater tertiary follicle development also appeared to demonstrate greater reproductive tract wet weight, presumably the result of elevated estrogen in circulation. These observations led to the hypothesis that gilts demonstrating tertiary follicle development earlier in life, assessed using vulva size as a proxy, achieve puberty earlier in life compared with counterparts of a similar age and weight that lack tertiary follicle development.

## MATERIALS AND METHODS

### Animals

This study was conducted at Iowa State University with animal procedures approved by the Iowa State University Animal Care and Use Committee. One hundred and sixty crossbred (Yorkshire × Landrace × Duroc) gilts of a similar age (65 ± 2 d) were selected and housed in pens of 4 and provided ad libitum access to feed and water for the project duration. Five animals were removed from the study due to lameness and/or receiving treatments for sickness leaving 155 animals used for the study.

### Data, Blood, and Tissue Collection

On postnatal days 75, 85, 95, 105, and 115 of age (PND) gilts were weighed and vulva measurements collected. Vulva length (VL) and vulva width (VW) measures were obtained using Ultra Tech digital calipers (General Tools, Secaucus, NJ) and recorded in millimeters. Vulva area was later calculated by multiplying the VL by VW. At each PND time point, 10 gilts were randomly selected and sacrificed. The reproductive tract including the uterus and ovaries were harvested immediately following euthanasia. The reproductive tract, including the broad ligament from the cervix to the oviducts and after ovarian removal at the mesovarium, was weighed and recorded. Ovaries were visually evaluated to quantitatively assess the number and size of tertiary follicles present also using digital calipers.

### Puberty Detection

A subset of gilts (*n* = 105) received daily boar exposure from 126 to 200 d of age to detect age at first estrus. Gilts received a daily minimum of 60 min of indirect (i.e., fence-line) exposure and 10 min of direct (physical contact) exposure to 1 of 2 sexually mature boars which were rotated daily between gilt pens. Physical contact was accomplished by allowing boar’s direct contact with gilts in the gilt pen and ally adjacent to gilt pens. During estrus detection, gilts were monitored for physical and behavioral estrus signs such as swollen and red vulva, interest in boars, and response to back pressure concomitant with boar exposure. The age at which gilts reached puberty onset was designated to be the first day at which behavioral estrus was observed.

### Statistical Analysis

Statistical Analysis System (SAS) University edition (SAS V9.4, SAS Institute Inc., Cary, NC) was used for all statistical analysis. Pearson correlations between physical measurements and age of puberty were determined using the PROC CORR procedure of SAS. Physical measurements were analyzed by puberty age groups as previously described as well as gilts achieving puberty before 200 d and those considered nonresponsive (puberty not detected within 200 d of age). Chi-square (χ ^2^) analyses were performed using the PROC-FREQ function within the SAS STAT software (SAS v9.4) to determine the association between body weight and vulva size with the ability to achieve puberty before or after 180 and 200 d of age.

## RESULTS

### Follicular and Reproductive Tract Development

**Figure 1.**
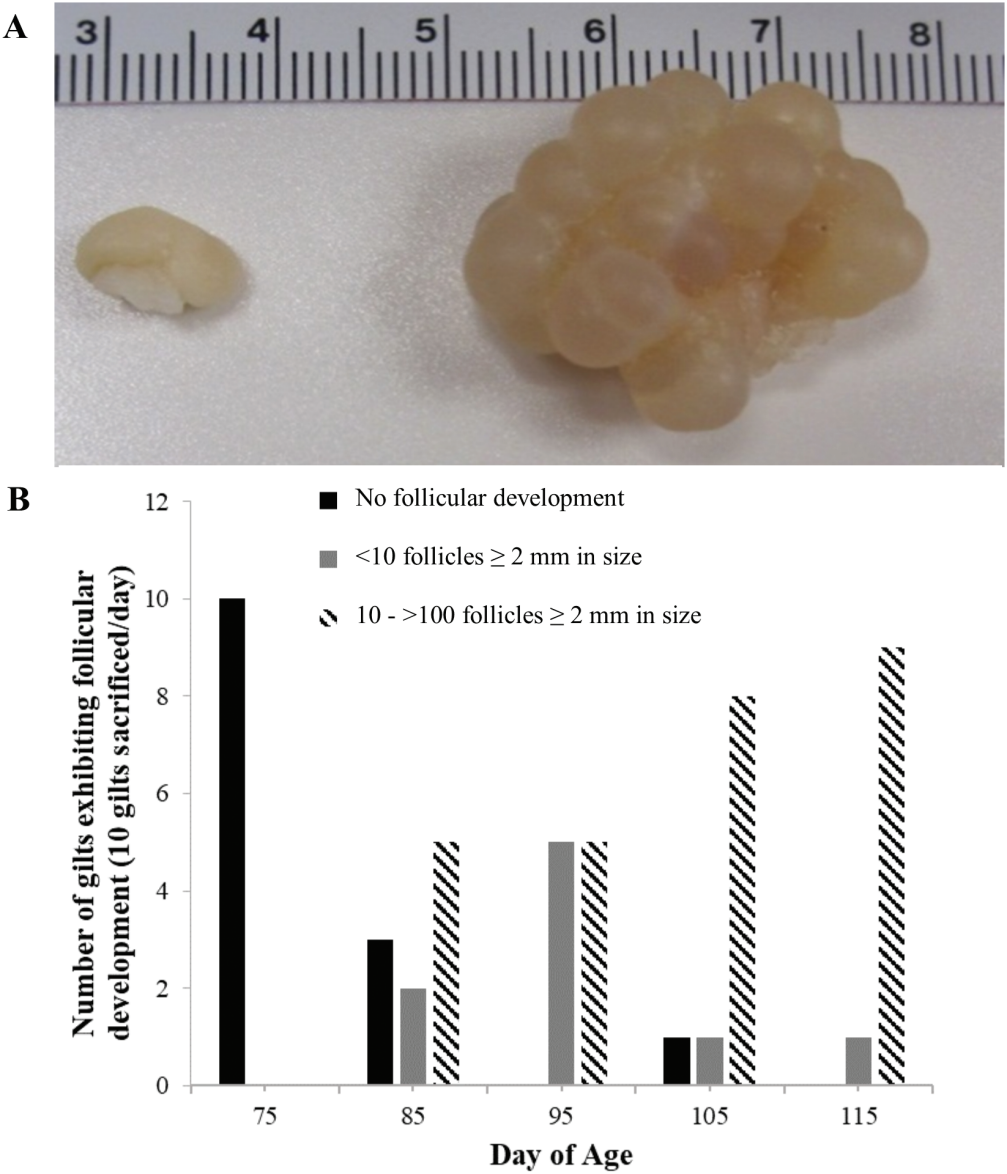
Representative image showing distinct variation in ovarian tertiary follicular development. (A) Ovaries collected from gilts of similar age (98 ± 4 d of age). Left—undeveloped ovary; right—ovary containing tertiary follicles. (B) Variation in follicular development in gilts exists between postnatal days 75, 85, 95, 105, and 115. As gilts progressed in age, follicular development increased. On postnatal day 75, all ovaries lacked tertiary follicles. Variation in tertiary follicular development was greatest between postnatal days 85 and 95. By postnatal day 115, all gilts had some form of follicular development with the majority of ovaries having tertiary follicles. The black bars represent the number of gilts lacking tertiary follicle development. Gray bars represent the number of gilts having a total of 1 to 10 tertiary follicles ≥ 2 mm in diameter. Striped bars represent the number of gilts having a total of 10 to greater than 100 tertiary follicles ≥ 2 mm in diameter.

**Figure 2.**
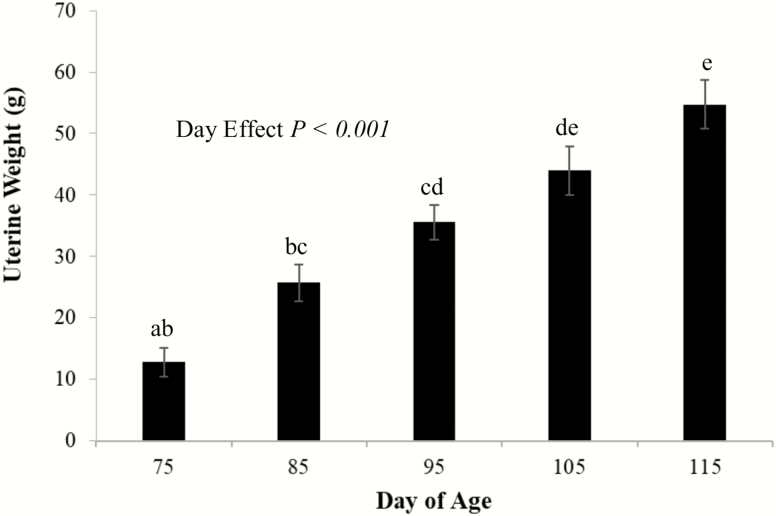
Average uterine weight of sacrificed gilts on postnatal days 75, 85, 95, 105, and 115. As gilts progressed in age, uterine weight increased across postnatal days 75 to 115. The increased weight of the reproductive tract is temporally associated with increased ovarian activity. Different superscripts represent statistical significance (*P* < 0.05).

Distinct variation in the number of measurable follicles was notable in gilt ovaries at each time point (Fig. 1). There was complete lack of follicular activity at PND 75 but all ovaries collected from PND 115 gilts had follicular activity (Fig. 1). Concomitant with increased follicular development, uterine weights increased (*P* < 0.05; Fig. 2) with increasing age: 12.8 ± 2.4, 25.7 ± 3.0, 35.6 ± 2.8, 43.9 ± 4.0, and 54.7 ± 3.9 g on PND 75, 85, 95, 105, and 115, respectively.

### Variation in Age of Puberty Onset and Body Weight Prior to Puberty

**Figure 3.**
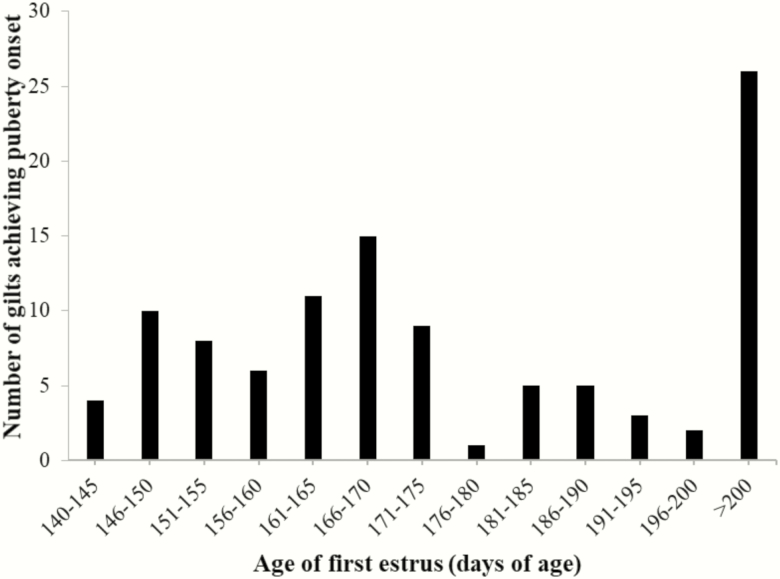
Distribution of gilts achieving puberty onset by 200 d of age. The first gilt achieved puberty on postnatal day 140 and boar exposure ended on postnatal day 200. Boar exposure and daily heat detection was initiated on postnatal day 129.

**Figure 4.**
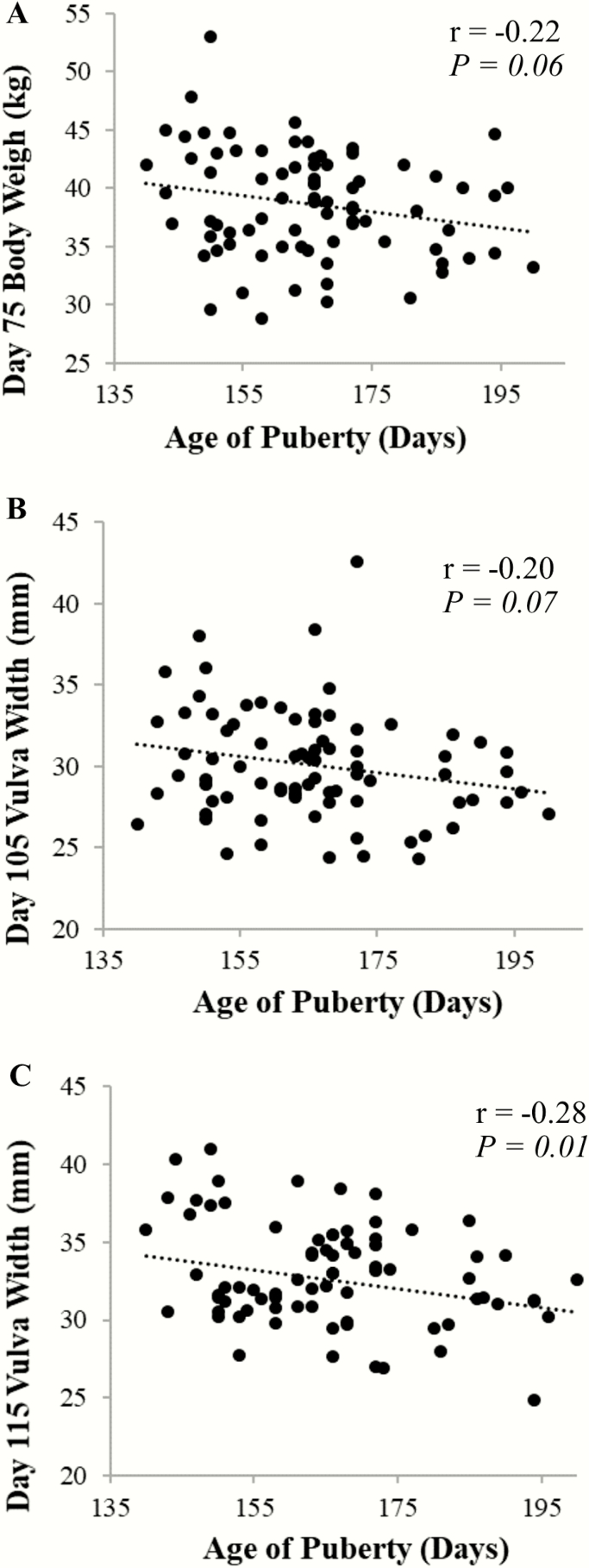
Relationship of growth parameters with age at puberty onset. A negative correlation exists between body weight on postnatal day 75 (A), vulva width on postnatal day 105 (B), and vulva width on postnatal day 115 (C) and age at puberty onset from those gilts achieving their first estrus by 200 d of age (A, *r* = −0.217, *P* = 0.055; B, *r* = −0.20, *P* = 0.07; C, *r* = −0.28, *P* = 0.01).

Puberty onset was recorded as the age when the first behavioral estrus was observed for each gilt. The earliest behavioral estrus identified was on PND 140 and 79 gilts demonstrated behavioral estrus by 200 d of age, while 26 gilts did not achieve behavioral estrus within the timeframe monitored (Fig. 3). The average age at puberty onset for the gilts achieving estrus was 165 d of age. The BW of the gilts used for estrus detection increased across the 5 PND time points (*P* < 0.05). The average BW was 38.6 ± 0.5, 49.1 ± 0.6, 57.8 ± 0.7, 68.1 ± 0.7, and 78.8 ± 0.8 kg at PND 75, 85, 95, 105, and 115, respectively (Table 1). The chi-square (χ ^2^) analyses indicated that there was no significant association between BW and puberty onset by either 180 or 200 d of age (χ ^2^ > 0.05; data not shown). However, of the gilts that demonstrated estrus behavior by PND 200, the correlation between BW at PND 75 and the age of puberty onset was detected (*r* = −0.22, *P* = 0.05; Table 1 and Fig. 4A) suggesting that of the gilts achieving puberty by 200 d of age, gilts with higher BW at PND 75 may have a slightly greater ability to achieve puberty earlier.

**Table 1.** Relationship between body weight during prepubertal growth and age at puberty onset

PND^1^	*N* ^2^	*r*-value^3^	*P*-value^3^	BW ± SEM^4^
75	79	−0.22	0.05	38.6 ± 0.5
85	79	−0.14	0.23	49.1 ± 0.6
95	78	−0.14	0.22	57.8 ± 0.7
105	79	−0.07	0.55	68.1 ± 0.7
115	79	−0.10	0.37	78.8 ± 0.8

^1^PND = postnatal day of age.

^2^Number of crossbred (Yorkshire × Landrace × Duroc) animals in analysis.

^3^Pearson correlation between body weight and age at puberty onset.

^4^Body weight mean ± SE.

### Relationship of Puberty Onset With Vulva Development prior to Puberty

Vulva width, VL, and VA increased over the 5 PND time points (*P* < 0.05). The average gilt VW were 22.8 ± 0.5, 24.7 ± 0.4, 27.8 ± 0.4, 30.1 ± 0.4, and 33.0 ± 0.4 mm at PND 75, 85, 95, 105, and 115, respectively (Table 2). The χ ^2^ values for VW on PND 75, 85, 95, 105, and 115 were 0.56, 0.74, 0.07, 0.24, and 0.84, respectively, suggesting that VW on PND 95 was marginally informative when predicating puberty onset prior to PND 200. However, of the gilts that demonstrated estrus behavior, there was tendency for correlation between VW and the age at first estrus at PND 105 (*P* = 0.07; Fig. 4B), and this reached significance at PND 115 (*P* = 0.01; Fig. 4C). This data suggest that between PND 105 and 115, gilts with larger VW have a greater probability to achieve puberty at an earlier age compared with gilts with smaller VW at the same age.

**Table 2.** Relationship between vulva width during prepubertal growth and age at puberty onset

PND^1^	*N* ^2^	*r*-value^3^	*P*-value^3^	VW ± SEM^4^
75	79	−0.08	0.47	22.8 ± 0.5
85	79	−0.12	0.30	24.7 ± 0.4
95	78	−0.12	0.31	27.8 ± 0.4
105	79	−0.20	0.07	30.1 ± 0.4
115	78	−0.28	0.01	33.0 ± 0.4

^1^PND = postnatal day of age.

^2^Number of crossbred (Yorkshire × Landrace × Duroc) animals in analysis.

^3^Pearson correlation between vulva width (VW; mm) and age at puberty onset.

^4^Vulva width mean ± SE (mm).

The average for VL from the 5 PND time points was 26.2 ± 0.6, 27.7 ± 0.4, 31.3 ± 0.5, 34.1 ± 0.5, and 38.2 ± 0.5 mm, respectively. The VL measure lacked correlation with age of puberty onset (*P* > 0.05; Table 3). The χ ^2^ values for VL on PND 75, 85, 95, 105, and 115 were 0.97, 0.93, 0.10, 0.69, and 0.47, respectively, suggesting that VL on PND 95 tended to be predictive when determining whether or not a gilt would achieve estrus prior to PND 200. The VA (VW × VL) average for PND 75, 85, 95, 105, and 115 were 612.6 ± 24.6, 694.0 ± 21.2, 879.9 ± 22.7, 1,036.8 ± 26.4, and 1,269.5 ± 28.0 mm^2^, respectively (Table 4). The χ ^2^ values for VA on PND 75, 85, 95, 105, and 115 were 0.56, 0.90, 0.10, 0.39, and 0.64, respectively, indicating that VA on PND 95 was marginally predictive in determining whether a gilt achieves estrus prior to PND 200. There was no correlation (*P* > 0.05) between VA and age at first estrus.

**Table 3.** Relationship between vulva length during prepubertal growth and age at puberty onset

PND^1^	*N* ^2^	*r*-value^3^	*P*-value^3^	VL ± SEM^4^
75	79	−0.06	0.58	26.2 ± 0.6
85	79	−0.01	0.96	27.7 ± 0.4
95	78	−0.07	0.57	31.3 ± 0.5
105	79	−0.04	0.70	34.1 ± 0.5
115	78	−0.04	0.73	38.2 ± 0.5

^1^PND = postnatal day of age.

^2^Number of gilts in analysis.

^3^Pearson correlation between vulva length (VL; mm) and age at puberty onset.

^4^Vulva length mean ± SE (mm).

**Table 4.** Relationship between vulva area during prepubertal growth and age at puberty onset

PND^1^	*N* ^2^	*r*-value^3^	*P*-value^3^	VA ± SEM^4^
75	79	−0.08	0.51	612.6 ± 24.6
85	79	−0.07	0.57	694.0 ± 21.2
95	78	−0.12	0.30	879.9 ± 22.7
105	79	−0.14	0.24	1036.8 ± 26.4
115	78	−0.17	0.14	1269.5 ± 28.0

^1^PND = postnatal day of age.

^2^Number of gilts in analysis.

^3^Pearson correlation between vulva area (VA; mm^2^) and age at puberty onset.

^4^Vulva area mean ± SE (mm^2^).

### Vulva Size as a Tool to Segregate Gilts Based on Potential to Achieve Puberty

**Figure 5.**
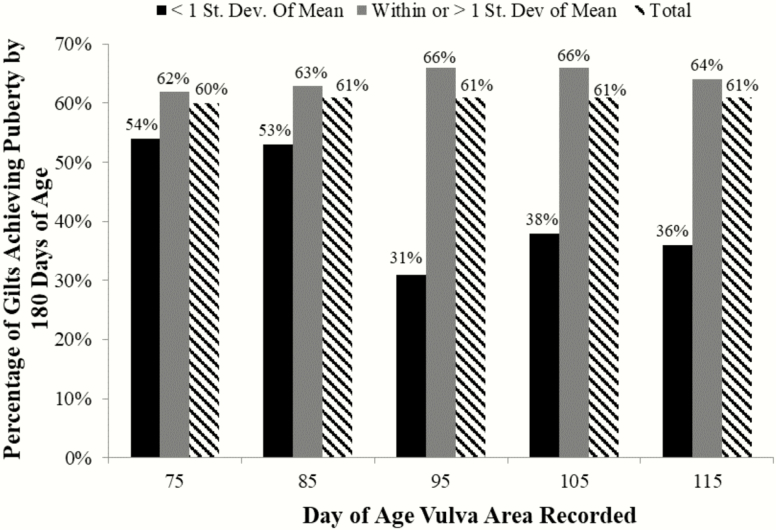
Percentage of gilts achieving estrus by 180 d of age on each postnatal day for a given vulva area. Gilts were grouped based on their calculated vulva area (VA) on the given day of age (X-axis). Black bars represent the percentage of gilts whose VA was less than 1 SD from the mean and achieved estrus prior to 180 d of age. The gray bars represent the percentage of gilts achieving estrus by PND 180 whose VA was within or greater than 1 SD from the mean. Striped bars represent the percentage of all gilts that achieved estrus by 180 d of age. A greater percentage of gilts with an average or above average vulva area on PND 95, 105, or 115 achieve puberty by 180 d of age than those gilts with a vulva area that is less than 1 SD from the mean.

**Figure 6.**
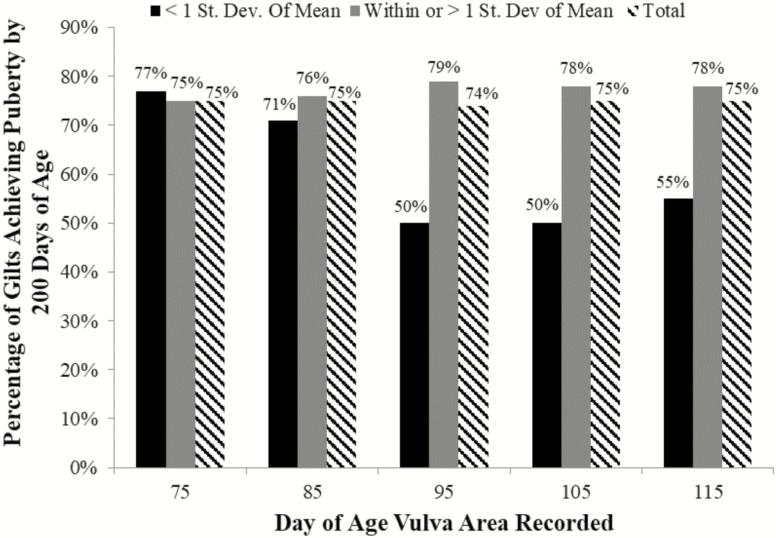
Percentage of gilts achieving estrus by 200 d of age on each postnatal day for a given vulva area. Gilts were grouped based on the calculated vulva area (VA) on the given day of age (X-axis). Black bars represent the percentage of gilts whose VA was less than 1 SD from the mean and achieved estrus prior to 200 d of age. The gray bars represent the percentage of gilts achieving estrus by postnatal day 200 whose VA was within or greater than 1 SD from the mean. Striped bars represent the percentage of all gilts that achieved estrus by 200 d of age. A greater percentage of gilts with an average or above average vulva area on postnatal days 95, 105, or 115 achieve puberty by 200 d of age than those gilts with a vulva area that is less than 1 SD from the mean.

To determine the practicality of selecting gilts based on VA prior to puberty, gilts were retrospectively grouped, based on VA, into 2 groups (below average or average/above average) for each PND that vulva measurements were recorded. Below average represented all gilts whose VA was smaller than 1 SD below the mean and average/above average included gilts with a VA within or greater than 1 SD from the mean. Of the gilts assigned to below average, 54%, 53%, 31%, 38%, and 36% achieved puberty by 180 d of age whereas 62%, 63%, 66%, 66%, and 64% of gilts assigned average/above average achieved puberty by 180 d of age when vulva measurements were made on PND 75, 85, 95, 105, and 115, respectively (Fig. 5). This result was consistent with determining the ability of gilts to achieve their first estrus by 200 d of age when grouped by their VA on different PNDs of development. Of the gilts designated below average, 77%, 71%, 50%, 50%, and 55% achieved puberty by 200 d of age, whereas 75%, 76%, 79%, 78%, and 78% of gilts designated average/above average achieved the same milestone when vulva size designations were assigned based on measurements taken on PND 75, 85, 95, 105, and 115 (Fig. 6). Collectively, when classifying gilts based on vulva size, 20% to 30% fewer gilts achieved estrus by PND 180 or 200 when their VA was considered below average on days 95, 105, or 115 (Figs. 5 and 6).

## DISCUSSION

The onset of puberty is a point in development when metabolic, endocrine, and physical factors are associated with achieving sexual maturation. Gilts make up a substantial portion of the inventory of the US commercial swine breeding herd, thus the time when they can be successfully mated, their ability to produce a litter, and remain productive for subsequent parities are all considerations greatly affecting overall herd reproductive performance and profitability. The age at puberty onset is in part controlled by individual animal genetics and can be variable in the time of developmental onset (Zak et al., 2017). Patterson et al. (2010) reported that neither gilt age or (BW) growth alone are useful predicators of age of puberty onset, but did identify the age of puberty as a useful indicator for a sow’s ability to produce at least 3 parities. Our previous findings identified a developmental timeframe within which variation in ovarian follicular activity and presumably ovarian functionality occurred (Pearce et al., 2013). Thus, the objectives of this study were to identify a developmental time point when variation in ovarian development exists, using vulva assessment as a bioassay proxy and to determine whether a relationship between prepubertal vulva development and age of puberty exits.

Rozeboom et al. (1995) reported a relationship in the timing of sexual maturation and growth in reaching a threshold to achieve puberty onset. This is in agreement with work by Le Cozler et al. (1998) that through restriction of feed, and subsequently growth, the timing at which a female reaches puberty can be delayed. Foxcroft et al. (1996) suggested that age of puberty onset in a female is reached well after the female has reached the threshold of growth needed to undergo sexual maturation. In this study, vulva measurements were conducted on PND 75, 85, 95, 105, and 115 gilts. A subset of gilts euthanized at each time point verified the variation in follicular activity across gilts at each age. This provided the opportunity to identify a specific timeline at which prepubertal gilts begin follicular development and to identify variation between gilts. A timeline of growth and development over 40 d was assessed and identified variation in follicular activity progressing from complete absence of tertiary follicular activity at PND 75 to demonstrable tertiary follicular activity on PND 115. This provided a temporal map to pinpoint a time at which ovarian biological activity could be compared with an observable and measurable phenotype in the form of VW, VL, and VA. This bioassay approach that serves as a potential proxy of ovarian activity has utility for pork producers to identify gilts with the greatest probability to undergo their first estrus at an earlier age than their counterparts.

We determined that the average age at estrus onset for gilts that demonstrated estrus within 200 d of age was PND 165. These results are consistent with Rozeboom et al. (1995), who reported the average age at puberty in a group of gilts reared with ad libitum feed intake was PND 172.5 with a SD of 23.4 d. Similar reports from Young et al. (1990) and Zimmerman et al. (2000) demonstrated an average age at puberty was PND 167.2 and PND 168, respectively. Although multiple factors can contribute to the age of puberty, such as age at the onset of puberty, we had approximately 25% of the gilts fail to achieve puberty by 200 d of age, which is not inconsistent with other literature (Patterson et al., 2002b; Calderón Díaz et al., 2017).

Kummer et al. (2009) suggested gilts with greater growth rates are more likely to have a decreased age at puberty when compared to slower growing cohorts. This study evaluated BW utility for as a puberty onset prediction and determined that utilizing BW on PNDs 75 to 115 was not useful at predicting whether gilts would achieve their first estrus by 200 d of age. However, BW on PND 75 was correlated with the age of estrus among gilts achieving puberty by 200 d of age, meaning that heavier gilts on PND 75 may have a higher likelihood to achieve estrus earlier when compared to lighter weight, age-matched gilts also achieving puberty by 200 d of age.

Vulva size was utilized as a proxy for increased follicular activity, as the increased presence of tertiary follicles associated with larger vulvas during PND 95 to 105 ostensibly induces the observed reproductive tract growth through elevated estrogen production. Based on these data, follicular activity during prepubertal gilt development begins between PND 75 and 85. Subsequently, PND 95 to 115 represents a useful time period of follicular development variation amongst a gilt cohort and is most noted when examining vulva development. To evaluate the effectiveness of using vulva size at a specific stage of prepubertal development in separating gilts into groups with different potential to achieve puberty by 200 d of age, we assessed using VA to stratify gilts into 2 groups based on the measurements collected at each of PND 75, 85, 95, 105, and 115. Interestingly, the percentage of gilts achieving puberty by 180 or 200 d of age was similar between gilts that had below average VA compared with gilts with average/above average VA when vulva assessments were done on PND 75 or 85. However, approximately 20% to 30% fewer gilts with below average VA on PND 95, 105, or 115 were able to achieve estrus by 180 or 200 d of age compared with their counterparts with average or above average VA (Figs. 5 and 6). Taken together, this approach could be an effective strategy to identify gilts with greatest reproductive potential, reducing costs, and improving production efficiency in the swine herd.

## SUMMARY AND CONCLUSION

Since the timing of puberty onset is highly variable and because it is labor intensive to identify it is usually not recorded in commercial swine production operations. Body weight at PND 75 and the changing vulva development spanning PND 95 to 115 may be useful predictors to help producers make replacement gilt selection decisions focused on increasing the number of gilts capable to achieving puberty onset by 180 or 200 d of age. The physiological development of the vulva may be a valuable selective tool for identifying replacement gilts that have a higher chance of entering the commercial breeding herd and becoming more productive as evidenced by a reducing the number of nonproductive days and increasing sow productive lifetime averages among the breeding herd female population.

## FUNDING

This project was supported with funding from the National Pork Board (Project #11-111).
